# Early maternal age and multiparity are associated to poor physical performance in middle-aged women from Northeast Brazil: a cross-sectional community based study

**DOI:** 10.1186/s12905-015-0214-1

**Published:** 2015-08-05

**Authors:** Saionara Maria Aires Câmara, Catherine Pirkle, Mayle Andrade Moreira, Mariana Carmem Apolinário Vieira, Afshin Vafaei, Álvaro Campos Cavalcanti Maciel

**Affiliations:** Department of Physiotherapy, Universidade Federal do Rio Grande do Norte, Avenida Senador Salgado Filho, S/N Caixa Postal 1524 - Campus Universitário - Lagoa Nova CEP, Natal, RN 59072-970 Brazil; Office of Public Health Studies, University of Hawaii-Manoa, 1960 East-West Road, Biomedical D104H, Honolulu, HI 96822-2319 USA; Department of Public Health Sciences, Carruthers Hall, Queen’s University, Kingston, Canada

**Keywords:** Life-course, Epidemiology, Aging, Adolescent pregnancy, Parity, Physical Performance

## Abstract

**Background:**

Adolescent childbirth and elevated parity are relatively common in middle and low-income countries and they may be related to the higher prevalence and earlier onset of physical decline documented in these settings, especially in women. The aim of this paper is to investigate whether reproductive history is associated with physical function in middle-aged women from Northeast Brazil.

**Methods:**

The relationship between poor physical performance (grip strength, gait speed and chair stand), early maternal age at first birth (<18 years old), and multiparity (≥3 children) was evaluated in a community sample of 473 women living in Parnamirim (Northeast Brazil). Linear regression models were used to examine the relationship of interest; in addition, mediation analyses were employed to assess indirect effects of obesity and family income.

**Results:**

Women who gave birth at less than 18 years of age took approximately 0.50 s longer to complete the chair stand test compared to women who gave birth at 18 years or older. Moreover, women who gave birth to < 3 children completed the chair stand test 0.42 s faster compared to those who had ≥ 3 children. The relation between reproductive history and physical performance was mediated by BMI. Reproductive history was not associated with performance in gait speed.

**Conclusions:**

This study provides evidence that adolescent childbirth and multiparity are related to worse physical performance in middle-aged women from a low income setting. Reproductive history may partially account for earlier physical decline and greater disability in women from lower income settings.

## Background

Decline in physical performance increases as individuals age, which constitutes an important public health concern. Grip strength, walking speed and time to rise from a chair are simple and objective measures of physical capability levels [1, 2]. Grip strength can provide a general indicator of overall fragility [1], while walking speed tests lower extremity function and time to rise from a chair assesses lower body strength [2]. Physical performance tests provide a marker of current health and predict subsequent health outcomes, such as disability, institutionalization and mortality in older populations [3].

At older ages, women on average tend to present lower physical performance than men, suggesting that gender or sex-linked dependent factors throughout life may influence physical performance [4]. In fact, a number of biological and social explanations are proposed to explain women’s relatively greater burden of physical decline and disability [5].

There is consistent evidence to support that social and economic adversity during the life course has strong effects on physical function [3], particularly among women in low income settings [6]. These women tend to start childbearing earlier; adolescent childbirth and elevated parity are relatively common in low and middle-income countries [7]. Women from lower-income settings tend to be more exposed to risks during childbirth and their reproductive histories may be at least partially related to the higher prevalence and earlier onset of physical decline documented in these settings, especially if early childbirth and higher parity affect future life opportunities [5, 7]. Childbearing may also contribute directly to accumulated physiological demands that over the long-term accelerate disability and death, because resources expended during childbirth cannot be used for later repair, in a process known as maternal depletion [8]. Alternatively, childbearing may indirectly contribute to physical decline through weight gain and greater overall body mass in later life [9, 10].

However, very little research has investigated a link between reproductive history and objective measures of physical function. As far as we know, only one study has investigated this relation and found that early maternal age at first birth is linked to lower physical performance in elderly from different settings [5].

Understanding health, behavioral and social factors that influence midlife performance may provide clues to the origins of frailty and disability in old age and the health of future elderly populations [11]. Thus, the aim of this paper is to investigate whether there is an association between reproductive history and physical function in middle-aged women from the Northeast of Brazil. We also explore whether any such associations are mediated by body mass index (BMI) and family income.

## Methods

This study took place in Parnamirim, a city in the Northeast of Brazil, which is located in Natal’s metropolitan region, the capital of Rio Grande do Norte state. This city has around 200,000 inhabitants, distributed across 123.5 km^2^, and is 100 % urbanized.

In this paper, we present the baseline data from an ongoing longitudinal research program examining physical performance in middle-aged women. The longitudinal study aims to analyze the influence of menopause and hormone levels on sarcopenia (muscle loss) and physical functioning. The present data were collected between April and November of 2013.

### Population and sample

The study population is composed of non-institutionalized women aged 40 to 65 years, living in Parnamirim. The baseline sample consists of 500 women recruited by advertisements placed in all primary care neighborhood centers across the city. Primary health care is universally funded by the Brazilian government through the Family Health Care program.

Exclusion criteria consisted of the following: neurological impairments and painful conditions, such as muscle and joint pain that might have compromised the physical performance measurement; being a smoker; or having had a double oophorectomy. In addition to the above criteria, for the present study, we excluded nulliparous women (*n* = 24) and women who did not complete the evaluation (*n* = 3). Nulliparous women were excluded because they may be different from parous women in ways that affect mortality [8]. Our final sample consisted of 473 women. This sample can be considered representative of the population of Parnamirim’s middle-aged women since the socioeconomic characteristics of the sample is similar to that of the wider population, according to the last census (2010).

### Ethics

All participants were informed of the objectives and procedures of the research study at first contact and signed a consent form. The study protocol received ethics approval by the Ethics and Research Committee of the Federal University of Rio Grande do Norte (approval number 387.737).

### Procedures

All women were assessed in a community center in Parnamirim by physiotherapists, trained by the principal investigator. The standardized protocols are described below.

### Physical performance

Physical performance was assessed with three tests: grip strength, gait speed and chair stands.

Grip strength: the dominant hand was evaluated with a Jamar® dynamometer in the second handle position [12]. The participant was positioned, as recommended by the American Society of Hand Therapists [13], seated with the shoulder fully adducted and neutrally rotated, elbow flexed at 90° and the forearm in a neutral position. The participant was requested to squeeze the dynamometer with maximal isometric effort without any other body movement, for five seconds. The test was performed three times, with one-minute intervals between measures. The mean of these three trials was used for analyses.

For gait speed, a 4-m walk at the participant’s usual pace was timed. The test was repeated twice with the faster of the two walks used. Gait speed was calculated in meters per second. For the ability to rise from a chair, participants were asked to stand up and sit down five times as quickly as possible with arms folded across their chests and were timed in seconds from the initial sitting position to the fifth standing position. Further details on the administration of these tests have been published in the original papers [14, 15].

### Reproductive history

Maternal age at first birth and parity were self-reported. We categorized age at first birth into less than 18 years old and 18 years or older, to separate women who gave birth as adolescents from those who gave birth as adults. After 18 years old, the musculoskeletal system of most women has reached complete development, they have accumulated sufficient nutritional reserves for pregnancy and the risk of obstetrical complication is reduced [16]. Thus, maternal age at first birth was categorized as: <18 years old, ≥18 years old. Parity was dichotomized in less than three births and three births or more. This cut-off was selected based on evidence that having three or more children is associated with coronary heart disease, stroke, and heart failure [17]. Finally, a composite variable was created to disentangle the influence of early age at first birth from having borne large numbers of children. Four groups were created: first childbirth before age 18 and three or more children; first childbirth before age 18 and less than three children; first childbirth at age 18 or older and three or more children; and first childbirth at age 18 or older and less than three children. Based on previous work, those women who gave birth as adults and had less than three children were considered as the lowest risk category [5, 18].

### Potential Confounders

Age was considered as a potential confounder because it is known to be associated with physical functioning [14, 19] and probably to reproductive history, since concerns about contraception and sexual education have increased in Brazil in recent decades [20].

Education was self-reported. We asked participants how many years of schooling they completed and then categorized the variable into: primary school (up to four years) and more than primary school (five years or more). Because girls may drop out of school as a result of pregnancy, and low educational attainment may be a consequence of adolescent childbearing, we dichotomized education at a point at which most women could not become pregnant.

To assess physical activity, the participants were asked about if they were currently taking part in sports, exercise, or other physical activities in their leisure time at least three times per week and for thirty minutes or more each time. Physical activity was categorized as yes or no.

Menopausal status was determined using the Stages of Reproductive Aging Workshop classification – STRAW [21]. Women were classified into three groups: premenopausal (regular menses), perimenopausal (irregular menses, with differences in cycle length over seven days or amenorrhea for up to one year) or postmenopausal (absence of menses for over one year or hysterectomy). We also created a variable to divide women who had undergone a hysterectomy or not. Having a hysterectomy has been associated with parity [22, 23] and may affect physical function, such as when there are complications associated with the surgery [24].

### Other variables

We explored potential intermediate variables on the pathway between reproductive exposures and physical performance: adulthood measures of socioeconomic position (family income) and BMI (body mass index). Using as a reference the Brazilian minimum monthly wage (MW), family income was categorized as less than three MW and three MW or more. The choice of three MW was based on what is considered to be a poverty threshold in the Northeastern Area of Brazil. BMI (kg/m^2^) was calculated from measured height (m) and weight (kg) and later categorized according to the international classification from the World Health Organization (WHO) as: 18.5 to 24.99 (normal weight), 25.00 to 29.99 (overweight), ≥30.00 (obese) [25]. It was hypothesized that if reproductive exposures were associated with physical performance, part of the association would be mediated by these variables [5, 18].

### Data Analysis

Analyses were carried out using SPSS software, version 20.0 (SPSS, Chicago, IL, USA). First, descriptive statistics for all variables were presented according to the variable maternal age at first birth and analyzed with analysis of variance (ANOVA) and post hoc Tukey test for continuous variables, and with Chi-square tests for comparison of proportions. Means and standard deviations of grip strength, gait speed and chair stands were presented for each category of the independent variables and compared using t-tests and ANOVA. Then multiple linear regression analyses were performed to model the effect of maternal age at first birth, parity and the composite variable for parous women on each physical performance measure, and adjusted for the covariates that had associations with physical performance with *p* < 0.20 in bivariate analysis (age, education, physical activity, menopausal status and hysterectomy).

**Fig. 1:**
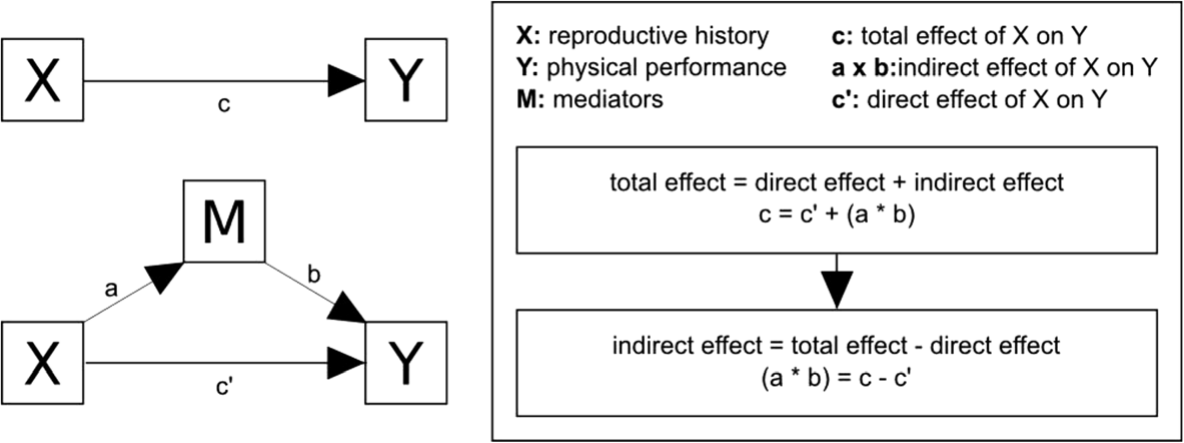
Strategy to analyze mediation factors between reproductive exposures and physical performance, using a model proposed by Preacher and Hayes (2004) [27]

We hypothesized that BMI and family income may mediate the relationship between the variables of reproductive history (age at first birth and parity) and the three physical performance measures. We adopted the strategy proposed by Hayes [26, 27] to explore potential indirect effects through these two potentially intermediate variables. According to this approach, the total effect of X on Y has two main components: a direct effect (c’) and an indirect effect through the mediator (s) of M. Testing mediation is based on exploring the statistically significance of the indirect effect (a*b) pathway in the direction predicted by the mediation hypothesis (Fig. 1). For each of the six relationships, we analyzed these two pathways in parallel as multiple mediator models adjusting for the same covariates used in regression analyses. To evaluate the significance of indirect effects, we used a nonparametric test to estimate 95 % confidence intervals (10,000 bootstrap sampling). The mediation analysis was performed using the Process Macros (http://www.afhayes.com/) developed for SPSS (IBM Corp., Armonk, NY, USA).

## Results

The women of this sample had their first child on average at 21.87 (±5.42; range 13 to 44) years old and had 2.86 (±1.53; range 1 to 12) children. Sample characteristics are presented according to maternal age at first birth (Table 1). There were no significant differences in the mean ages of participants according to when they had their first child. The proportions of participants within each category of menopausal status were relatively similar, as were the proportions of participants reporting hysterectomies. No significant differences were observed in self-reported physical activity. Women who had children before 18 years of age were significantly more likely to have a family income less than three MW compared to women who had children later. Likewise, women who had children as adolescents were significantly less educated than women who had children as adults. They also had more children over their lifetimes. Compared to women who had children as adults, those who had children at younger than 18 were nearly twice as likely to have 3 or more children. Finally, those women who had children as adolescents were more likely than the other group of women to be overweight or obese. For all categories of women, the majority were overweight or obese.

**Table 1:** Sample characteristics according to age at first birth (*N* = 473)

Variables	Before 18 years old (*n* = 102)	18 years old or more (*n* = 371)	*p* value
	n (%) or mean (SD)		
Age (years)	49.66 (5.54)	50.12 (5.58)	0.457^a^
Family income			0.005^b^
<3 MW	83 (81.4)	249 (67.1)	
≥ 3 MW	19 (18.6)	122 (32.9)	
Education			<0.001^b^
Primary school	41 (40.2)	70 (18.9)	
More than primary	61 (59.8)	301 (81.1)	
BMI (kg/m^2^)			0.034^b^
18.5-24.9 (normal)	17 (16.7)	82 (22.1)	
25.0-29.9 (overweight)	34 (33.3)	156 (42,1)	
≥30.0 (obese)	51 (50.0)	133 (35.8)	
Physical activity			0.779^b^
No	77 (75.5)	275 (74.1)	
Yes	25 (24.5)	96 (25.9)	
Parity			<0.001^b^
0-2 children	13 (12.7)	199 (53.6)	
3 or more	89 (87.3)	172 (46.4)	
Menopause Status*			0.158^b^
Premenopausal	26 (25.7)	76 (20.9)	
Perimenopausal	24 (23.5)	122 (33.6)	
Postmenopausal	51 (50.0)	165 (45.5)	
Hysterectomy			0.134^b^
Yes	24 (23.5)	64 (17.3)	
No	78 (76.5)	307 (82.7)	
Total	102 (20.5)	371 (74.6)	

MW minimum wages, *BMI* Body Mass Index

*10 women could not describe their pattern of menstruation

^a^p value for *t*-test

^b^p value for Chi-square test

Table 2 shows the descriptive statistics for physical performance according to the independent variables. On average, women who gave birth at less than 18 years of age had weaker grip strength, slower gait speeds and took more time to complete the chair stand test compared to the other categories of women, but the differences were statistically significant only for chair stand test. Parity was significantly associated with the grip strength and chair stand tests, with better performance for women with less than 3 births.

**Table 2:** Mean levels of physical performance according to covariates (*N* = 473)

Factors	Grip strength	Gait speed	Chair stands
	(Kgf) *	(m/s) ^†^	(s) ^††^
	Mean (SD)		
Total sample	26.01 (5.31)	0.987 (0.182)	10.20 (2.02)
Age at first birth			
Before 18 years old	25.39 (4.37)	0.979 (0.183)	10.55 (2.08)
18 years old or more	26.18 (5.53)	0.989 (0.182)	10.10 (2.00)
*p value* ^a^	0.180	0.636	0.050
Family income			
< 3 MW	25.43 (5.14)	0.977 (0.187)	10.35 (1.99)
≥ 3 MW	27.35 (5.46)	1.010 (0.170)	9.83 (2.05)
*p value* ^a^	<0.001	0.075	0.011
Education			
Until primary school	25.01 (4.87)	0.954 (0.180)	10.39 (2.20)
More than primary	26.32 (5.41)	0.997 (0.182)	10.14 (1.96)
*p value* ^a^	0.024	0.031	0.255
BMI (kg/m^2^)			
18.5-24.9 (normal)	24.85 (4.76)	0.990 (0.180)	9.90 (1.60)
25.0-29.9 (overweight)	26.29 (5.43)	1.006 (0.186)	10.10 (2.15)
≥30.0 (obese)	26.34 (5.39)	0.965 (0.178)	10.45 (2.06)
*p value* ^b^	0.051	0.100	0.078
Physical activity			
No	25.79 (5.35)	0.974 (0.181)	10.33 (2.02)
Yes	26.63 (5.16)	1.023 (0.182)	9.82 (1.98)
*p value* ^a^	0.134	0.011	0.021
Parity			
< 3 children	26.70 (4.92)	0.100 (0.189)	9.94 (1.94)
≥ 3 children	25.45 (5.54)	0.975 (0.178)	10.41 (2.07)
*p value* ^a^	0.011	0.124	0.014
Menopausal Status			
Premenopausal	27.82 (5.88)	0.990 (0.157)	9.62 (1.75)
Perimenopausal	25.74 (5.22)	0.968 (0.170)	10.33 (1.83)
Postmenopausal	25.31 (4.97)	0.994 (0.193)	10.34 (2.22)
*p value* ^b^	<0.001^c^	0.382	0.010^c^
Hysterectomy			
Yes	26.18 (4.63)	1.029 (0.210)	10.46 (1.96)
No	25.95 (5.46)	0.977 (0.174)	10.14 (2.04)
*p value* ^a^	0.001	0.015	0.190

*Kgf* kilograms-force, *MW* minimum wages, *BMI* Body Mass Index

*Grip strength was evaluated with a Jamar® dynamometer. Greater scores indicate better grip strength. ^†^Gait was measured with a four meter walk at the participants usual speed. Greater measures indicate faster gate speed. ^††^Chair stands were evaluated by asking the participants to stand up and sit down as quickly as possible. Greater measures indicate longer times to complete the task

^a^p values for *t*-test. ^b^p valeus for ANOVA. ^c^premenopausal > perimenopausal; premenopausal > postmenopausal

For the covariates, we observed that women who reported regular physical activity had faster gait speed and chair stand tests. Education was significantly associated with lower grip strength and gait speed measures. Premenopausal women had better grip measures and faster chair stands, while women who did *not* have a hysterectomy had lower grip strengths and slower gait speeds. Finally, for the intermediate variables, we observed that those with low incomes had significantly lower grip strengths, gait speed and took longer to complete the chair stand test. There was no significant association with BMI; however, mean scores of overweight and obese women were greater for both the grip measures and chair stand tests.

Tables 3 and 4 show the multiple linear regression results for each performance test by maternal age at first birth and parity, respectively. In model 1, we present the unadjusted model, in model 2 the results are adjusted for age and education and in model 3 the variables of physical activity, menopausal status and hysterectomy are included. The chair stand measure was statistically different among categories of age at first birth and parity in the fully adjusted model (model 3). Women who had their first birth before 18 years old (Table 3) or who had 3 children or more (Table 4) presented worse performance in the chair stand test after adjustment for the covariates. It took the women who gave birth at less than 18 years of age approximately 0.50 s longer to complete the chair stand test compared to women who gave birth at 18 years or older. Moreover, women who gave birth to less than 3 children completed the chair stand test 0.42 s faster compared to those who had 3 children or more. The relation between grip strength and the variables of reproductive history was not significant in the adjusted models. We did not observe an association between maternal age at first birth or parity with gait speed.

**Table 3:** Multiple regression models for performance tests (grip strength, gait speed and chair stands) by age at first birth (*N* = 473)

	Model 1		Model 2		Model 3	
Grip strength (Kgf)	β (95 % CI)	*p* value	β (95 % CI)	*p* value	β (95 % CI)	*p* value
Before 18y	−0.796 (-1.963: 0.370)	0.180	−0.729 (-1.905: 0.447)	0.224	−0.821 (-2.018: 0.375)	0.178
18y or more	0		0		0	
Gait speed (m/s)						
Before 18y	−0.010 (-0.050: 0.031)	0.636	−0.002 (-0.043: 0.040)	0.934	−0.005 (-0.046: 0.036)	0.821
18y or more	0		0		0	
Chair Stands (s)						
Before 18y	0.452 (0.000: 0.905)	0.050	0.453 (-0.009: 0.915)	0.055	0.492 (0.024: 0.961)	0.040
18y or more	0		0		0	

Model 1 : Unadjusted. Model 2 : Adjusted for age and education. Model 3 : Adjusted for age, education, physical activity, menopausal status and hysterectomy

**Table 4:** Multiple regression models for performance tests (grip strength, gait speed and chair stand) by parity (*N* = 473)

	Model 1		Model 2		Model 3	
Grip strength (Kgf)	β (95 % CI)	*p* value	β (95 % CI)	*p* value	β (95 % CI)	*p* value
1-2 children	1.248 (0.287 : 2.210)	0.011	0.782 (-0.210 : 1.775)	0.122	0.817 (-0.188 : 1.822)	0.111
3 or more	0		0		0	
Gait speed (m/s)						
1-2 children	0.026 (-0.007 : 0.059)	0.124	0.016 (-0.019 : 0.051)	0.367	0.011 (-0.023 : 0.045)	0.531
3 or more	0		0		0	
Chair stands (s)						
1-2 children	−0.468 (-0.841 : -0.096)	0.014	−0.387 (-0.777 : 0.004)	0.052	−0.422 (-0.817 : -0.027)	0.036
3 or more	0		0		0	

Model 1: Unadjusted. Model 2: Adjusted for age and education. Model 3: Adjusted for age, education, physical activity, menopausal status and hysterectomy

Table 5 presents the linear regression models for the composite age at first birth/parity variable. Women who gave birth before 18 years old and also had 3 or more children presented worse performance in grip strength and chair stand tests in the unadjusted models. After adjusting for the covariates (model 3), giving birth at less than 18 years of age and having 3 or more children was significantly associated with only the chair stand test. On average, these women took 0.66 s more to complete the chair stand test, compared to women who gave birth as adults and only had 1 or 2 children. There was also a borderline association with the chair stand test for women who gave birth as adults and had 3 or more children (β 0.379; p 0.089). For grip strength, there was a borderline association in the adjusted model (model 3) (*p* = 0.055) for women who gave birth at 18 years of age or less and had 3 or more children. On average, these women had -1.35 kgf less than women who gave birth as adults and had 1-2 children.

**Table 5:** Multiple regression models for performance tests (grip strength, gait speed and chair stand) by the composite age at first birth/ parity variable (*N* = 473)

	Model 1		Model 2		Model 3	
Grip strength (Kgf)	β (95 % CI)	*p* value	β (95 % CI)	*p* value	β (95 % CI)	*p* value
First child before 18 and ≥3 children	−1.613 (-2.938 : -0.289)	0.017	−1.236 (-2.599 : 0.127)	0.076	−1.350 (-2.730 : 0.030)	0.055
First child before 18 and <3 children	1.438 (-1.533 : 4.409)	0.342	0.726 (-2.236 : 3.688)	0.630	0.993 (-2.083 : 4.070)	0.526
First child at 18y or more and ≥3 children	−0.924 (-2.007 : 0.160)	0.095	−0.504 (-1.602 : 0.594)	0.367	−0.478 (-1.591 : 0.635)	0.399
First child at 18y or more and <3 children	0		0		0	
Gait speed (m/s)						
First child before 18 and ≥3 children	−0.030 (-0.076 : 0.016)	0.194	−0.018 (-0.066 : 0.030)	0.471	−0.016 (-0.064 : 0.031)	0.495
First child before 18 and <3 children	0.067 (-0.036 : 0.170)	0.201	0.065 (-0.039 : 0.168)	0.221	0.064 (-0.041 : 0.169)	0.233
First child at 18y or more and ≥3 children	−0.018 (-0.055 : 0.020)	0.356	−0.010 (-0.048 : 0.029)	0.615	−0.003 (-0.041 : 0.035)	0.871
First child at 18y or more and <3 children	0		0		0	
Chair stands (s)						
First child before 18 and ≥3 children	0.672 (0.157 : 1.187)	0.011	0.618 (0.081 : 1.156)	0.024	0.655 (0.113 : 1.197)	0.018
First child before 18 and <3 children	0.396 (-0.739 : 1.532)	0.493	0.573 (-0.571 : 1.718)	0.326	0.829 (-0.356 : 2.014)	0.170
First child at 18y or more and ≥3 children	0.401 (-0.019 : 0.821)	0.061	0.324 (-0.107 : 0.756)	0.140	0.379 (-0.058 : 0.815)	0.089
First child at 18y or more and <3 children	0		0		0	

Model 1: Unadjusted. Model 2: Adjusted for age and education. Model 3: Adjusted for age, education, physical activity, menopausal status and hysterectomy

Table 6 shows the multiple mediator models for age at first birth and parity on the three physical performance tests. Of the two potential mediators, only BMI was a significant pathway. To assess the robustness of our models we performed two additional sensitivity analyses. In a serial mediation model, we tested the significance of the pathway of Reproductive History > Family Income > BMI > Physical Function. We also constructed mediator models adjusting only for age and education. In all, only BMI was a significant mediator (results can be obtained upon request).

**Table 6:** Multiple mediator models showing total, direct and indirect effects of age at first birth and parity on physical performance tests

	Grip Strength			Gait speed			Chair Stands		
	Estimate	SE	95 % CI	Estimate	SE	95 % CI	Estimate	SE	95 % CI
First Birth									
Total Effect	0.7821	0.6100	−0.4168: 1.9809	0.0048	0.0208	−0.0361: 0.0456	−0.4796 ^*^	0.2390	−0.9494: -0.0099
Direct Effect	0.8180	0.6057	−0.3724: 2.0085	−0.0025	0.0209	−0.0436: 0.0386	−0.3780	0.2403	−0.8502: 0.0942
Indirect Effects									
Total	−0.0360	0.1474	−0.3727: 0.2329	0.0073^†^	0.0040	0.0012: 0.0177	−0.1017^†^	0.0473	−0.2235:-0.0258
Family income	0.1680	0.0850	0.0333: 0.3756	0.0025	0.0021	−0.0004:0.0080	−0.0356	0.0258	−0.1007:0.0024
BMI	−0.2039^†^	0.1159	−0.5426:-0.0475	0.0049^†^	0.0033	0.0003:0.0150	−0.0661^†^	0.0395	−0.1778:-0.0114
Parity									
Total Effect	0.8082	0.1156	−0.1993:1.8157	0.0105	0.0175	−0.0238:0.0449	−0.4172^*^	0.2016	−0.8134: -0.0209
Direct Effect	0.8374	0.5070	−0.1590:1.8339	0.0057	0.0175	−0.0287:0.0402	−0.3459	0.2018	−0.7426:0.0508
Indirect Effects									
Total	−0.0293	0.1287	−0.3033:0.2052	0.0048^†^	0.0032	0.0002: 0.0134	−0.0712^†^	0.0402	−0.1639:-0.0126
Family income	0.1110	0.0914	−0.0168:0.3581	0.0017	0.0020	−0.0004: 0.0085	−0.0205	0.0229	−0.0872:0.0045
BMI	−0.1403^†^	0.0879	−0.3992:-0.0162	0.0031^†^	0.0024	0.0000: 0.0103	−0.0507^†^	0.0308	−0.1347:-0.0059

Data adjusted by age, education, physical activity, menopausal status and hysterectomy. *Significant p < 0.05 for total and direct effects

^†^Significant indirect effect as demonstrated by 95%CI of 10,000 bootstrapping

## Discussion

The results of this study show that variables of reproductive history (parity and maternal age at first birth) are associated with physical performance in middle-aged women, especially when evaluated with the chair stand test. Women who gave birth before 18 years of age had lower mean chair stand test scores, even after extensive adjustment for potential confounders. Similar findings were observed among women who had three children or more. It was difficult to dissociate early childbearing from multi-parity, because most women who gave birth during adolescence also had three or more children. Among those women who gave birth to their first child as adults, we observed a borderline association between having three or more children and longer chair stand test times, but the effect estimate was almost half the size of that for women who gave birth as teenagers and had three or more children (β = 0.38 versus 0.66). Finally, we observed that part of the association between reproductive variables and physical performance may be mediated through greater BMI.

To our knowledge, only one previous study [5] investigated the relation between age at first birth and objectively measured physical performance, but in an older sample of women (65-75 years old). It showed that the effect of early maternal age on physical function was stronger for elderly women from Canada and Albania than women from Latin American sites. It was suggested that many of the Latin American women may have died before the study began, which is consistent with research demonstrating disproportionate mortality in those with lower socioeconomic advantage [28]. Chair standing is one of the most difficult physical function tests to complete and it may be indicative of early disablement. If women who gave birth as adolescents are already beginning the disablement process in middle-age, then they may disproportionately die before older age. This in turn may hamper detection of physical function differences in older age, because those surviving are not representative of the sub-population of women who gave birth as adolescents.

Several social and physiological pathways may explain the association between early maternal age at first birth and poor physical performance. Pregnancy in low income settings combined with poor nutrition may reduce physiologic reserves [8, 16], making women more susceptible to losses of mobility in middle and older age. The association between early maternal age at first birth and longer mean times to complete the chair stands may also reflect maternal morbidities that occurred early in the life-course. Adolescent childbirth is a risk factor for severe pelvic injury, including obstetric fistula, which is still prevalent in resource poor settings [29–31]. Pelvic organ prolapse, which is common in women over 50 years of age [32], is consistently associated with reproductive history [33], as is urinary incontinence [34]. The risk of pelvic organ prolapse significantly increases with increasing parity, especially for vaginal childbirth [33]. Other potential obstetrical risk factors for pelvic organ prolapse include: early age at first birth, forceps delivery, and prolonged 2^nd^ stage of labour [33]. In this study, parity was strongly associated with maternal age at first birth. Moreover, when many of these women were first giving birth, access to high quality maternal health services was low [35]. It is possible that the chair stand assessment is sensitive to the physical discomfort associated with injuries to the pelvic region originating from childbirth. Moreover, early birth before complete skeletal development and multiple births may damage the bones and ligaments around the hips and pelvis, affecting movements such as standing up from a chair later in life [5].

Early maternal age at first birth and high parity favor accumulation of excess weight over a lifetime, which is consistent with our finding of an indirect pathway between the reproductive variables and longer chair stand times. Data from both men and women in United States suggests that parenthood is associated with accelerated weight gain over time, when compared to those with no children [9]. Moreover, this research demonstrated that both early first childbirth and increasing parity are associated with greater body weight; however, the association with parity was only observed in women [9]. Similarly, in a British sample of middle-aged men and women, having had one’s first child as an adolescent was associated with significantly greater BMI in later life [10]. In our sample, fifty percent of the women who gave birth before 18 were considered obese according to BMI classification, against 35 % of those who gave birth after 18. The physiological adaptations during pregnancy and childbirth such as insulin resistance, dyslipidemia, fat accretion and inflammation may persist after giving birth [5] and over time, contribute to the development of chronic disease and loss of physical function [5].

Early maternal age at first birth and multiple parity may also contribute to poverty later in life. Particularly, early maternal age at first birth may jeopardize the social and economic opportunities available to a woman due to incomplete education, low skilled occupations and fewer employment opportunities. Previous studies have reported associations between socioeconomic position during the life course and health in adulthood, with consistent evidence that the socioeconomically disadvantaged have poorer physical function than the more advantaged [3, 6, 11, 36, 37]. In our study, however, we did not observe mediation attributable to current socio-economic position, as measured with family income.

### Strengths and limitations

As far as we know, this is the first study investigating the association between objective measures of physical performance and reproductive history in a population of middle-aged women. Although women in this sample were recruited through advertisements in primary health centers, their socioeconomic characteristics are similar to other samples of community-based studies in the area [6, 38], and this sample also presents similar socioeconomic characteristics to the population of Parnamirim’s women according to the last census data.

Because our sample contained middle-aged women, the variance for grip strength and gait speed may not have been large enough to detect small changes with a relatively small sample. Indeed, the inability or slowness to rise from a chair seems to appear earlier than slowness in regular walking in older populations. Thus, it is not surprising to observe that changes in chair stand times are more variable than changes in gait speed around midlife.

We do not have information about childhood socioeconomic position of the sample and the reproductive exposures of adolescent childbearing and/or multiparity could be partial consequences of low childhood socioeconomic position. Thus, reproductive history may be on the causal pathway between childhood socioeconomic position and later adult health. In other words, adversity during a woman’s childhood may initiate a cascade of events, including adolescent childbearing, multiparity and high BMI that ultimately contributes to lowered physical function in middle age. More investigation into the causal pathways linking childhood and adolescent events to poorer adult health is warranted.

Finally, although some information was collected by self-reporting and bias can occur for those with lower education, self-reported questionnaires are a typical method employed in the literature. Furthermore, objective and validated measures of physical performance were used. Use of such measures is unusual in a cohort of middle-aged adults; they are typically used in cohorts of elderly individuals.

## Conclusions

The present study shows that middle-aged women who gave birth before 18 years old and/or had 3 or more children presented worse physical performance. Higher BMI may mediate this association. The results highlight the importance of health promotion policies and practices that target young women from middle and low income settings. Providing sex education and contraception for these women may reduce the negative life-long consequences of early pregnancy and high parity. Child leave policies and educational programs for teen mothers may also mitigate the negative socioeconomic consequences of early childbearing and having large families, with positive influences on physical functioning later in life.

## Abbreviations

STRAW: Stages of Reproductive Aging Workshop classification
BMI: Body mass index
MW: Monthly wage
WHO: World Health Organization
